# The Financial Costs and Effects on the Well-Being of Nursing Students’ Professional Experience Placements: A Cross-Sectional Comparative Study of Urban and Rural Experiences

**DOI:** 10.3390/ijerph22121848

**Published:** 2025-12-10

**Authors:** Sandra Coe, Annette Marlow, Sarah J. Prior, Carey Mather

**Affiliations:** 1 Rural Clinical School, University of Tasmania, Burnie, TAS 7320, Australia; 2 Tasmanian School of Medicine, University of Tasmania, Hobart, TAS 7000, Australia; annettemarlow23@gmail.com; 3 Tasmanian School of Medicine, University of Tasmania, Burnie, TAS 7320, Australia; 4 Paramedicine and Healthcare Sciences, School of Nursing, Charles Sturt University, Wagga Wagga, NSW 2678, Australia; carey.mather@utas.edu.au; 5 School of Nursing, University of Tasmania, Launceston, TAS 7250, Australia

**Keywords:** undergraduate nursing, student placement, work-integrated learning, rural education, financial hardship

## Abstract

There is sustained research interest examining what constitutes positive and effective professional experience placements. However, few studies have investigated the financial costs of placements for health profession students. This study bridges the research gap by analysing the financial impacts of placements reported by nursing students in 2018/2019. The study analyses feedback from nursing students at the University of Tasmania, using a comparative lens analysing the costs for urban and rural placements (in this paper the term ‘rural’ includes regional, rural, remote and very remote). The results indicate students undertaking rural placements in Tasmania may be better supported than students with urban placements. This finding may be a result of the package of support provided by UTAS for its pre-registration nursing students when undertaking placements in rural locations.

## 1. Introduction

Professional experience placements (‘placements’) are an important and necessary aspect of undergraduate nursing education in Australia. Placements assist students to synthesise theory into practice in real-world settings, develop clinical skills and facilitate their socialisation into nursing practice [1]. The Australian Nursing and Midwifery Accreditation Council (ANMAC) mandates undergraduate nursing students complete at least 800 h of placement experience to meet accreditation requirements [2]. As a compulsory component of course accreditation in Australia, placements exhaust nursing students’ time and their resources [3]. If students are undertaking paid employment alongside their studies, this is adversely impacted when they are relocated for placement or are required to undertake shifts that do not accord with their usual employment hours, creating financial strain that can affect their well-being [4].

As a significant component of nursing education, placements have attracted considerable research interest as researchers seek to determine what constitutes effective placement learning experiences, and to improve the quality of such experiences. Current research predominantly focuses on supervision practices and student experiences [5,6] and includes models for training and educating supervisors to improve supervision practices during placements [7]; the practicalities of employing facilitators to improve supervision during placements [8,9,10,11], along with the exploration of students’ perspectives on their placement experiences to determine good supervision practices [12,13,14,15]. Despite sustained research interest in placements, the financial costs students incur for participating in this activity have been largely overlooked. When the financial impacts of placements are examined, researchers generally focus on the specific costs of initiatives rather than the costs students incur undertaking these activities [16,17].

Student perspectives on the financial cost of placement have only recently attracted the research gaze and are a developing sub-field of research. Grant-Smith and de Zwaan [18] explored students’ coping strategies to manage the financial stress of placements and identified four themes that connected placements and financial stress—reduced ability to undertake paid work during placement, costs associated with placement, other placement factors and students’ personal coping strategies [18]. Schofield, Keane and Fletcher et al. [19] examined the financial costs of rural placements for medical, nursing, and allied health students. They found nursing students worked longer and were more financially disadvantaged than other health students. Wray and McCall [20] investigated medical, nursing, and allied health students’ financial concerns associated with placements and found that placements caused students “acute financial hardship” (p. 978). Usher, Fagan, Brown et al. [3] undertook a national study of nursing students in Australia and found that many students were unable to continue paid employment during professional experience placements (PEP). They found the triggers for financial impacts during placement are loss of income, transport costs and placement relocation costs [3]. Generally, research suggests the financial costs of placements are higher for students undertaking rural placements compared to those with urban placements [21,22,23].

Tasmania is an island state in Australia and is predominantly classified by the Modified Monash Model (MMM) [24] as rural, with only two urban centres approximately 200 kilometres apart (Hobart and Launceston). The University of Tasmania (UTAS) is the sole university on the island and also has a presence on the Australian mainland with a campus in Sydney in New South Wales (NSW). UTAS nursing students in Tasmania undertake placements across the state (a mix of urban and rural regions), whilst those in Sydney primarily undertake placements within the city’s regions. The characteristics of Tasmania as a predominantly rural state mean that rural regions are located in relatively close proximity to the two urban locations of Hobart and Launceston. Thus, rural placements do not necessarily mean students have to travel long distances to placement locations. Further, students can study nursing from all UTAS Tasmanian campuses, including the campus at Burnie which is classified as regional.

As the only university in Tasmania, UTAS has a strong interest in developing and promoting rural health workforce development and receives funding from the Commonwealth Department of Health to expand and support rural placements under the Rural Health Multidisciplinary Training Program (RHMTP) [25]. This funding support is restricted to regional, rural, and remote placements. Within its support packages, UTAS provides nursing students with subsidised accommodation for rural placements within Tasmania. It also provides additional support, such as the Whole of Community Facilitators who directly support students during rural placements [8]. At times, students on rural placements also receive financial supplements—such as a rural placement allowance. The supplement and accommodation support are intended to reduce the financial burden of rural placements for students and to encourage and promote rural experiences. Students with urban placements do not receive the package of support offered to students with rural placements.

The aim of this study was to investigate the financial impacts for UTAS nursing students, and to compare those costs between urban and rural placements. This research is guided by the following questions:

i. What are the financial costs nursing students incur participating in compulsory placements?
ii. Are there differences between these costs for urban and rural placements?

## 2. Materials and Methods

This study uses data collected as part of a national study that surveyed nursing students across nine universities within five Australian states [3]. Respondents were enrolled in a Bachelor- or Masters-level pre-registration nursing course between 1 October 2018 and 31 October 2019 and nominated their educational institution (N = 2359) [3]. The survey consisted of 28 questions that investigated the location of and travel to the most recent placement, and students’ employment, accommodation, financial support, expenses and debts, and financial strain pertaining to placements. Placement preference was scored on a five-point Likert Scale (1 = never to 5 = always). Financial strain/hardship items were scored on a five-point Likert scale (1 = strongly disagree to 5 = strongly agree). Scores < 3 indicated disagreement, and scores > 3 indicated agreement. Financial strain/hardship responses were collapsed into disagree (strongly disagree and disagree) and agree (agree and strongly agree) for the purpose of reporting, and all cost data was reported in AUD (2021) [3]. The project received ethics approval from the UTAS Human Research Ethics Committee (H0017908). Ethics approval granted the data use for both the pooled national survey and the specific UTAS cohort data.

### 2.1. Data Collection

All survey respondents identified as UTAS students were extracted from the national study by its lead investigator and imported into a single Microsoft Excel (Excel) spreadsheet, with each row capturing the full survey data for each respondent. The survey was anonymous, and it was not possible to remove responses once submitted. Participants consented by submitting the survey which was outlined in the information sheet and preamble on the questionnaire prior to the start of the online questionnaire.

### 2.2. Data Analysis

Data was analysed in Microsoft Excel as a whole and stratified by urban or rural placement using percentages and frequencies. Analyses undertaken on the whole data were replicated on the stratified samples for comparison purposes with results presented descriptively as percentages. Free text responses were analysed and categorised using a general inductive approach [26]. Two authors (CM, AM) developed codes and independently coded the data. Coding was then compared and developed into broader like-categories, discussed and revised as needed. Where useful, percentages were calculated to identify themes.

## 3. Results

Eighty-six percent of respondents completed the survey, 91% completed two-thirds of the survey, and 5% completed only the demographic questions. There were no personal demographic questions in the survey. Respondents were primarily enrolled in an undergraduate nursing course (98%). One UTAS respondent identified as a Masters-level student, and seven did not indicate their course level. Fifty-five percent had urban placements and 45% had rural placements. Of rural placements, 93 were regional, 71 were rural, and 5 were remote placements using the MMM, combined in this paper as ‘rural’.

Nursing students at UTAS generally undertake five placements across their pre-registration degree programme—a two-week placement in the first year, two three-week placements in the second year, and two placements of six and seven weeks each in the third year [27]. A greater percentage of respondents were in the second and third year of educational preparation (Table 1).

Three overarching categories were derived from the survey and interview data: Financial strain and well-being, Placement debt and Employment impacts. Each category is described in detail below.

### 3.1. Financial Strain and Well-Being

Survey questions primarily focused on various aspects of costs and well-being. Four scale questions probed the nexus between financial strain and well-being during placement as shown in Table 2.

Table 2 shows that, overwhelmingly, respondents’ placement experiences were impacted by financial strain with 75% of respondents agreeing to the statement about experiencing financial hardship, although not all respondents indicated this financial strain caused stress (65%). Even fewer indicated financial strain impacted their enjoyment of their most recent placement (45%), although more respondents indicated it impacted their health and well-being (57%).

Stratifying results by placement region suggested rural placements generated slightly more financial strain for respondents than urban placements, with rural respondents at a slightly higher percentage for questions 25 (78% rural, 72% urban) and 26 (65% rural, 64% urban). However, financial strain impacted more respondents with urban placements than respondents with rural placements based on percentages for questions 27 (50% urban, 41% rural) and 28 (58% urban, 55% rural). Results suggest that although respondents with rural placements experienced financial strain, it did not necessarily detract from a positive placement experience. In contrast, urban placement respondents had lower rates of financial hardship and stress, but their enjoyment of placement was impacted more than those students with rural placements.

### 3.2. Placement Debt

Multiple survey questions gathered data about placement incurred debt (Table 3). A total of 32%of respondents indicated they incurred debt due to their latest placement with ‘loan from family’ (57%) and ‘credit card debt’ (34%), the primary forms of debt, with ‘personal loan’ debt 13% and ‘other’ debt 7%. Analysis of free text comments indicated ‘other’ debt included household bills (groceries, housing payments, telephone invoices, insurance), suggesting respondents struggled to pay their usual general living expenses during placement. Stratified analysis showed more urban placement respondents (33%) incurred debt during PEP than rural placement counterparts (25%). These results contradict the results for questions 25 and 26.

Respondents were asked to estimate their overall costs for attendance at their most recent placement with survey responses sought in AUD 500 increments from zero to AUD 5000. Exact cost amounts could also be entered into the categories of accommodation, transport, parking, meals, childcare, lost wages, and other. With most costs below AUD 1000 higher costs were consolidated into a ‘AUD 1000 and above’ category. Zero-cost and non-responses were omitted from this analysis.

For all categories, except transport, percentage rates for respondents with urban placements were higher than those with rural placements. These results suggest the support UTAS provides for rural placements may improve some placement-related costs for respondents.

**Figure 1:**
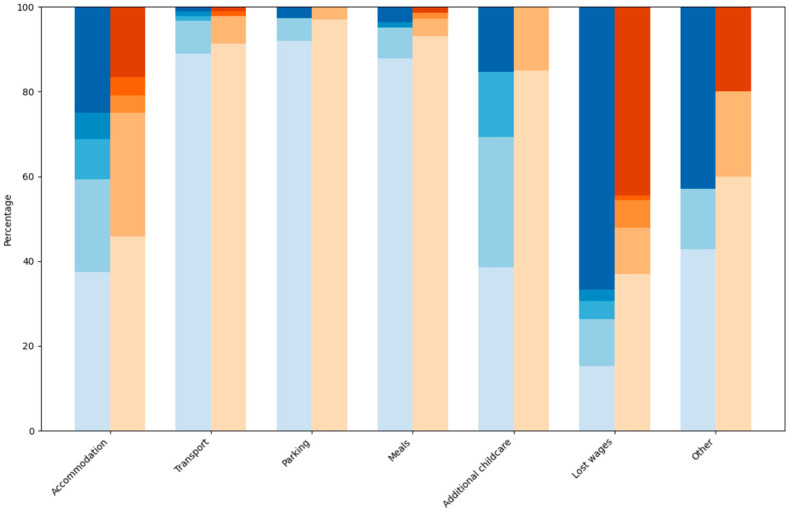
Reported overall costs for attendance at most recent placement, as percentage.

Figure 1 shows respondents’ reported expenses by cost categories, with most reported costs below AUD 500, except for lost wages. The blue lines indicate the expenses for urban participants and the orange lines show the rural participants expenses. Almost half of each group had lost wages of AUD 1000 or above (48% for urban and 41% for rural placement respondents). The percentage of respondents with urban placements was higher than rural placement respondents for all costs groupings in the expenses category of AUD 1000 and above. More respondents with rural placements than urban placements had transport costs below AUD 500. For the expenses category up to AUD 499, a higher percentage of urban placement respondents had costs for accommodation, parking, and meals. The percentage of rural placement respondents was higher for transport, additional childcare and lost wages. These results indicate that, in Tasmania, the financial costs of placements were higher for urban placement respondents than for rural placement respondents.

The survey allowed respondents to report weekly accommodation costs, although not all respondents provided this information. Table 4 records this data for rural and urban respondents and indicated the accommodation costs for most rural placement respondents were under AUD 200 with 36% having zero costs and 52% less than AUD 200. For urban placement respondents only 13% had zero costs with 76% incurring costs below AUD 300. Respondent comments suggest that rural placement students without accommodation either elected not to use the university accommodation provided or were unaware of these accommodation options.

Further survey responses about travel and transportation indicated 78% of respondents had access to a vehicle during placement. Eighty-seven percent of rural placement respondents had access to a vehicle during placement compared to 71% of urban placement respondents. A total of 82% of urban placement respondents could access public transport, whilst only 55% of rural placement respondents (55%). More rural placement respondents had no access to public transport (36%) compared to urban placement respondents (13%). For respondents without access to public transport, comments indicated there was no bus service, public transport schedules did not fit with placement shifts, or there was no public transport in the placement region.

For both urban and rural placements most respondents spent one hour travelling to their latest placement and travelling less than 50 km. More urban (25%) than rural (11%) placement respondents spent two hours or more travelling to placement. Sixty-nine percent of rural placement respondents travelled less than 50 km for their placement.

Most respondents (67%) had access to accommodation, although 29% rural placement respondents, and 3% urban placement respondents did not. For respondents without access to accommodation, most did not report problems finding accommodation. More than half of each group stayed in their own homes. For both groups, 26% stayed with either family or friends. Sixteen percent of urban placement respondents rented accommodation, Air BnBs or hotels. It is unclear from the survey data how rental accommodation was defined. For most rural placement respondents their accommodation costs were under AUD 200 with 36% having zero costs and 52% less than AUD 200. For urban placement respondents only 13% had zero costs with 76% incurring costs below AUD 300.

Respondents were asked about their home-related expenses during placement. They were able to select multiple responses from prescriptive categories. More than half indicated expenses such as rent, electricity, internet, and general household expenses. Only 12% indicated childcare and child-related expenses. Other expenses were internet, electricity, and rent. A fifth of all respondents indicated mortgage expenses were a cost. Without additional demographic data, further analysis of expenses by respondents’ family or caring responsibilities was unavailable.

Respondents were asked to nominate strategies used to reduce costs from prescriptive responses. Most indicated they chose locations where accommodation was available with family and/or friends. For rural placement respondents, selecting locations with university accommodation available was a common strategy. Urban placement respondents selected locations with no- or low-cost parking available. Respondents also travelled with other students to reduce costs. Respondents’ free-text comments indicated they employed a range of cost-cutting or saving strategies to reduce the financial impacts of placements. These strategies included being frugal, saving for placement, and working extra hours:

“*be more financially frugal. Keep living expenses as low as possible*” (urban placement respondent).

“*Try to budget for meals each day and go without certain things to afford the extra petrol*” (urban placement respondent).

“*bin dove for free food, reduced socialising and recreational activities due to budget*” (urban placement respondent).

“*Work extra hours outside placement to accrue additional leave*” (rural placement respondent).

Some respondents stated they had no control over placement locations and therefore could not control costs:

“*I didn’t have the option to travel with anyone else, nor do I have the option to select areas to cut costs*” (rural placement respondent).

“*Does not apply as we have no choice where we are sent*” (rural placement respondent).

### 3.3. Employment Impacts

Most respondents were casually employed (67%), although more rural placement respondents were employed (81%) than urban placement respondents (76%). Only 2% of respondents had full-time employment. Fifteen percent of respondents indicated their employment was not affected by their latest placement.

Of the respondents that indicated their employment was impacted by placement, 49% indicated they were unable to work during their latest placement. Twenty-nine percent reduced work hours during PEP, and 17% took leave to cover their placement period. The free-text comments revealed the impact PEP had on respondents’ employment:

“*Could not work for two weeks, was offered shifts on weekend but was too tired and run down to be able to do them*” (rural placement respondent).

“*I had to use annual leave in order to have 1 day off per week, so 5 days of placement, 1 day of work and 1 day of annual leave as a day off*” (urban placement respondent).

“*I cannot do placement & take shifts at work with a toddler it is far too much*” (urban placement respondent).

Trying to maintain employment during placement meant some respondents worked continuously throughout the placement period:

“*I was unable to work my normal hours to support my financial needs. I was working on the weekends when I could which meant I was working 19 days straight (including unpaid placement)*” (rural placement respondent).

“*I had to work each weekend just to earn enough money to pay rent and eat as well as being on placement*” (urban placement respondent).

Reduced availability for paid work during placement impacted some respondents’ employment post-placement:

“*I was contacted to do many shifts even though I had notified them of my 6-week unavailability and this has also affected me getting shifts on my return back to my employment place as I have been seen to not to be an involved casual employee due to no shifts been undertaken in a 6 week period*” (rural placement respondent).

“*Reduced availability due to placement lead to reduced shifts after placement*” (rural placement respondent).

## 4. Discussion

This study aimed to investigate the financial impacts for UTAS nursing students who undertake placement, and to compare costs between urban and rural placements. The results indicated that respondents’ placement experiences were impacted by financial strain, and some indicated this also impacted their health and well-being, however this did not always cause stress or reduce their enjoyment of their most recent placement. Rural placements created slightly more financial strain for respondents than urban placements, which is consistent with Usher et al. [3] who found that nursing students in urban placements had fewer financial issues than those placed in regional areas. However, although rural placement respondents experienced financial strain, it did not necessarily detract from a positive placement experience. Overall, the results suggest UTAS students enjoyed their placements despite the financial challenges placements caused.

Results suggested respondents struggled to pay usual general living expenses during placement, most often due to reduced capacity for paid work during placement participation. Stratified analysis showed, contrary to other studies [4] more urban placement respondents incurred debt than rural placement counterparts during PEP. Accommodation, transportation, parking, meals, additional childcare, lost wages, and other general living costs all impacted respondents during PEP. This ‘placement poverty’ [4] has been described previously as significantly impacting nursing students across all areas and contributes to students using various strategies to cut costs. This includes choosing placement sites with no parking costs, or places where they are able to rely on family and friends for accommodation [28]. Further, in our study urban placements incurred more debt than rural placement respondents in all categories, except transport. In rural placements students can drive up to 400 km per day to avoid paying for accommodation [28,29]. Consistent with these findings more than half of all students in placement stayed in their own properties to avoid accommodation costs altogether.

Accommodation was the highest expense in urban placements as only 13% had zero costs with 76% incurring costs below AUD 300. It is unsurprising accommodation was a significant cost for urban placements, given the RHMTP provides accommodation support for rural placements. Further, given the current housing crisis facing most regions in Australia, particularly larger urban areas, accommodation impacts will become more significant for nursing students who have to relocate for PEP.

As UTAS provides, or subsidies, accommodation for nursing students undertaking rural placements in Tasmania, on sites close to all rural placement venues, accommodation costs were not as impactful for students with rural PEP. For most rural placement respondents their accommodation costs were under AUD 200, with 36% having zero costs and 52% less than AUD 200.

These results on accommodation diverge from the national study which found rural placement respondents had “*significantly more*” trouble acquiring accommodation than urban placement respondents [3]. The national study found sourcing accommodation was a major concern for regional students, with many students having to rent a flat or room in share houses, or staying in caravan parks, home stays and motels [3]. Rural accommodation was reported as being expensive because of the placement length and location, sometimes in a tourist area or during the school holidays [3].

Consistent with previous studies, other factors that contributed to excess costs for placement, in general, included wages, meals, parking, additional childcare [18] which were more often noted in urban settings in this study. Respondents estimated placement-related monetary costs to be less than AUD 500, although some suggested it was higher than AUD 1000.

With most respondents employed in some way, unsurprisingly, most indicated the placement affected their employment (56%). Almost half of students reported they were unable to work during their latest placement regardless of their placement location. This finding is consistent with other studies in nursing and allied health [1,2,3,4], and also the national study by Usher, Fagan, Brown et al. [3] who found placements adversely impacted students’ employment, with some losing work or feeling pressured by employers to work during placement periods. With more than half of all respondents indicating PEP affected their employment, this cost had the most financial impact for respondents. For some, the costs to employment are more widespread, adversely impacting their post-placement employment.

These results indicate that placements, regardless of where they occur, create debt for nursing students as they are unable to engage in paid employment to cover their everyday living expenses. Furthermore, for those students without access to a private vehicle, public transport timetabling generally does not accommodate hospital shifts. Respondents reported using various strategies to mitigate the cost burdens of placement. Some saved money before placement to cover their usual expenses during placement. Others used annual leave, reduced their living costs by living frugally, and made use of supported accommodation to reduce costs during placement.

In Tasmania, students with rural placements are supported with accommodation close to placement venues. This support is provided via the RHMTP funding, which does not support urban placements. Consequently, students with urban placements incur greater placement costs than those with rural placements. Although respondents thought rural placements created greater debt, the survey costs analysis indicated urban placements in Tasmania created more debt for respondents that rural placements. These results suggest the UTAS initiatives funded via the Commonwealth RHMTP to support students on rural placements offset some of the financial burdens of placement. Furthermore, recently, in response to the Australian Universities Accord Recommendation 14, the Commonwealth Prac Payment (CPP) was incepted in the 2024–2025 Australian Government Budget to support tertiary nursing, midwifery, social work or education students [30,31]. This payment acknowledges the placement poverty and now alleviates some of the financial burden of eligible pre-registration nursing students with a stipend during placements. The authors argue this funding is essential and recommend its continuation to support the development of the future rural nursing workforce.

### Limitations

The lack of personal demographic information and capacity to stratify the data in the national survey limited the analyses undertaken. More information about respondents’ personal and familial responsibilities would provide additional insight into the consequences of the financial impacts of PEP, particularly regarding relocation, carer responsibilities and across jurisdictions. It was also unclear how many respondents received their choice of placement location. It was also unknown how many respondents were aware of the accommodation and financial support provided by UTAS.

## 5. Conclusions

The findings of this study highlighted the financial impact of placements in urban and rural areas and that costs can be reduced by the provision of supported accommodation and financial supplements during rural placements. While some differences were noted between the urban and rural impact, it is recommended that further research be undertaken to delve into demographic factors influencing these outcomes. Further, ongoing financial support is essential for the development of the future nursing workforce, and particularly the rural workforce.

## Figures and Tables

**Table 1:** Length of placement for rural and urban locations.

	Rural (N = 158)		Urban (N = 207)	
	N	%	N	%
1 week (40 h)	2	1	1	0.5
2 weeks (80 h)	54	32	81	39
3 weeks (120 h/20 days)	2	1	2	1
4 weeks (160 h)	66	39	85	41
6 weeks (240 h)	33	20	38	18.5
9 weeks (360 h) (making up placement time)	1	1	0	0

**Table 2:** Scale question responses, by urban and rural placements, as percentages.

	Strongly Disagree	Somewhat Disagree	Neither Disagree or Agree	Somewhat Agree	Strongly Agree
Question—“I experienced financial hardship on my most recent clinical placement”					
All respondents (N = 320)	32 (10%)	15 (5%)	32 (10%)	121 (38%)	120 (37%)
Urban placement (N = 173)	16 (9%)	10 (6%)	23 (13%)	55 (32%)	69 (40%)
Rural placement (N = 147)	15 (10%)	4 (3%)	14 (9%)	66 (45%)	49 (33%)
Question—“My most recent clinical placement was stressful due to financial issues”					
All respondents (N = 319)	3 (10%)	25 (8%)	56 (18%)	123 (39%)	83 (26%)
Urban placement (N = 173)	16 (9%)	14 (8%)	32 (18%)	64 (36%)	48 (28%)
Rural placement (N = 146)	18 (12%)	10 (7%)	23 (16%)	60 (41%)	35 (24%)
Question—“It was difficult to enjoy my most recent clinical placement due to financial strain”					
All respondents (N = 319)	51 (16%)	51 (16%)	73 (23%)	93 (29%)	51 (16%)
Urban placement (N = 173)	26 (15%)	28 (16%)	33 (19%)	55 (32%)	31 (18%)
Rural placement (N = 146)	23 (16%)	25 (17%)	39 (27%)	39 (27%)	20 (14%)
Question—“The financial strain of my most recent clinical placement affected my health and well-being (exhaustion, stress, depression, etc.)”					
All respondents (N = 319)	51 (16%)	29 (9%)	57 (18%)	112 (35%)	70 (22%)
Urban placement (N = 173)	27 (16%)	17 (9%)	29 (17%)	57 (33%)	43 (25%)
Rural placement (N = 146)	25 (17%)	13 (9%)	28 (20%)	54 (37%)	26 (18%)

**Table 3:** Reported costs from most recent placement.

	N	%
**Number of respondents with debt, by placement**		
All respondents (N = 376)	**110**	**29**
Urban placement respondents (N = 207)	68	33
Rural placement respondents (N = 169)	42	25
**Accommodation**		
All respondents	**95**	**28**
Urban placement respondents	58	32
Rural placement respondents	37	24
**Transport**		
All respondents	**306**	**90**
Urban placement respondents	165	90
Rural placement respondents	144	92
**Parking**		
All respondents	**118**	**35**
Urban placement respondents	67	36
Rural placement respondents	51	32
**Meals**		
All respondents	**272**	**80**
Urban placement respondents	151	82
Rural placement respondents	115	73
**Additional childcare**		
All respondents	**38**	**11**
Urban placement respondents	23	13
Rural placement respondents	15	10
**Lost wages**		
All respondents	**246**	**72**
Urban placement respondents	134	73
Rural placement respondents	112	71
**Other**		
All respondents	**20**	**6**
Urban placement respondents	12	7
Rural placement respondents	8	5

**Table 4:** Reported weekly accommodation costs from most recent placement for rural and urban locations.

	Rural (N = 64)		Urban (N = 61)	
	N	%	N	%
AUD 0	22	36	8	13
AUD 1–AUD 100	18	29	15	24
AUD 101–AUD 200	14	23	21	34
AUD 201–AUD 300	4	7	11	18
AUD 301–AUD 400	1	2	3	5
AUD 401–AUD 500	2	3	4	6

## Data Availability

The data presented in this study are available on request from the corresponding author due to ethical approval.

## Abbreviations

The following abbreviations are used in this manuscript:

MMM	Modified Monash Model
PEP	Professional experience placements
RHMT	Rural Health Multidisciplinary Training
UTAS	University of Tasmania
